# Supplementary material for: Virtual Reality Combined with Artificial Intelligence (VR-AI) Reduces Hot Flashes and Improves Psychological Well-Being in Women with Breast and Ovarian Cancer: A Pilot Study
Source: Healthcare (Basel). 2022 Nov 11;10(11):2261. doi: 10.3390/healthcare10112261 (PMC9690775; doi:10.3390/healthcare10112261)
Supplement: Supplementary file 1 [file healthcare-10-02261-s001.zip › healthcare-1943731-supplementary.pdf]

Bubble Health LTD. Technology, Data and Algorithms

The Bubble Health ecosystem comprises four main elements:

1. Bubble Mobile Application
   Available on both Android and iOS devices, Bubble allow users to place their mobile device within a VR headset and enter the Bubble 3D VR experience, where they can choose between different guided scenarios for the treatment of hot flashes, preprocedure anxiety, PTSD, depression, etc.
   Created using Unity, Bubble gathers feedback from the users, both from the choices the user used within the experience and the biofeedback from the hardware headset (such as tilting of the head, jerking motion, etc.) and sends the data into the Bubble server backend where it is processed.
2. Bubble Server Backend
   The server backend is the throbbing heart of the Bubble experience. It is the place where all info is gathered from all individual users and calculated across the user domain to determine the best experiences, changing the visual graphics of the app accordingly.
   Moreover, for each individual user, there is a learning curve that determines what type of experience is best and what should be added or removed in the next session of the user-app experience. The rate at which the viewer moves within the 3D VR experience, the rate of interactions, ambient sounds and guidance dialog change as more info is gathered on the user, thus creating a unique user experience that fits the need of the individual user.
   All gathered data are encrypted and kept safe and segregated from other users to ensure users' anonymity.
   The AI learning mechanism is based on clustered LDI (linear, differential, integral), where specific orders are provided to the mobile app to quickly change and adapt the user's ongoing experience to ultimately lower anxiety. The AI can change, for example, the color scheme of the scenario within the VR world and

the sounds around the user (for example, the sound and rate of breathing, the sounds of footsteps in the snow), objects can move slower or faster and items can pop up to interact with the user.

The database on the server side of the app is constantly evolving, adding new experiences both graphical and text based so as to keep the user engaged while fitting the “right” session to each user.

3. Luna Chatbot

The Luna chatbot is a simple mobile application created in Java for Android devices and in React for iOS devices. The main purpose of the Luna chatbot is to interact with the users outside the main Bubble app by means of a simple chat with the Bubble AI, placed in the backend.

The main purpose of Luna is to guide the user, through a series of well-scripted scenarios and questions, to understanding when and where to use the app, including useful tips and tricks for between sessions of the main app. Luna uses a complex decision tree where the user is provided multiple choice response options where each given answer leads to the next relevant question and so on until the chatbot’s AI understands how fruitful the last interaction with the main app was and suggests the next best time to use the app as well as set a reminder for the user to continue using the app when needed.

In addition, the chatbot is meant to answer simple questions regarding the usage of the main Bubble app, resolve technical issues, remind the user to use the main app, gather written feedback from the user and assess user satisfaction.

The Luna chatbot is mostly server side based, where the backend AI constantly learns the user's habits and provides new and innovative ways of communicating with the user. The user is randomly provided with new and different text messages that keep the conversation going and prevent leaving the chatbot due to repetitive answers.

The server side's AI is mostly Java based with a simple DB to handle the texts and user replies.

4. Hardware VR Headset

Designed but never implemented, the hardware headset was supposed to have a built-in heart rate variability (HRV) sensor, breathing sensor, skin conductivity sensor (to detect sweating) and mini camera for eye movement detection.

The main idea was to gather biofeedback from the users and integrate it within the 3D VR experience, changing what the user sees in correlation to how the body of the user reacts at that moment.

Data would have been processed by both the mobile app and the backend server.

Data would also have been gathered across all the user domain to analyze and provide medial insight into how the human body reacts to changing VR scenarios.
